# Predictors of therapeutic failure in GH and prolactin co-secreting pituitary adenomas

**DOI:** 10.1530/EC-25-0103

**Published:** 2025-07-15

**Authors:** Marta Araujo-Castro, Betina Biagetti, Edelmiro Menéndez, Iría Novoa-Testa, Fernando Cordido, Víctor Rodríguez Berrocal, Eider Pascual-Corrales, Fernando Guerrero-Pérez, Almudena Vicente, Rogelio García-Centeno, Laura González, María Dolores Ollero García, Ana Irigaray Echarri, María Dolores Moure Rodríguez, Cristina Novo-Rodríguez, María Calatayud, Rocío Villar-Taibo, Ignacio Bernabéu, Cristina Alvarez-Escola, Carmen Tenorio Jimenéz, Pablo Abellán-Galiana, Eva Venegas, Inmaculada González-Molero, Pedro Iglesias, Concepción Blanco, Fernando Vidal-Ostos De Lara, María Paz de Miguel Novoa, Elena López-Mezquita Torres, Felicia Hanzu, Cristina Lamas, Silvia Aznar Rodríguez, Anna Aulinas, José María Recio, María Dolores Aviles-Pérez, Miguel Antonio Sampedro Núñez, Rosa Camara, Miguel Paja Fano, Carmen Fajardo, Luís Cardoso, Pedro Marques, Elena Martínez-Sáez, Ignacio Ruz-Caracuel, Mónica Marazuela, Manel Puig-Domingo

**Affiliations:** ^1^ Endocrinology & Nutrition Department, Hospital Universitario Ramón y Cajal, Madrid, Spain; ^2^ Instituto de Investigación Biomédica Ramón y Cajal (IRYCIS), Madrid, Spain; ^3^ Endocrinology & Nutrition Department, Hospital Universitario Vall de Hebrón CIBERER group 747, Barcelona, Spain; ^4^ Endocrinology & Nutrition Department, Hospital Universitario Central de Asturias, Asturias, Spain; ^5^ Instituto de Investigación Sanitaria del Principado de Asturias (ISPA), Asturias, Spain; ^6^ Endocrinology & Nutrition Department, Hospital Universitario A Coruña & Universidad de A Coruña, A Coruña, Spain; ^7^ Neurosurgery Department, Hospital Universitario Ramón y Cajal, Madrid, Spain; ^8^ Endocrinology & Nutrition Department, Hospital Universitario de Bellvitge, Cataluña L'Hospitalet de Llobregat, Spain; ^9^ Endocrinology & Nutrition Department, Hospital Universitario de Toledo, Toledo, Spain; ^10^ Endocrinology & Nutrition Department, Hospital Universitario Gregorio Marañón, Madrid, Spain; ^11^ Endocrinology & Nutrition Department, Hospital Universitario Navarra, Pamplona, Spain; ^12^ Endocrinology & Nutrition Department, Hospital Universitario de Cruces, Bilbao, Spain; ^13^ Endocrinology & Nutrition Department, Hospital Universitario Virgen de las Nieves, Granada, Spain; ^14^ Endocrinology & Nutrition Department, Hospital Universitario Doce de Octubre, Madrid, Spain; ^15^ Endocrinology & Nutrition Department, Hospital Universitario de Santiago de Compostela, Madrid, Spain; ^16^ Endocrinology & Nutrition Department, Hospital Universitario La Paz, Madrid, Spain; ^17^ Endocrinology & Nutrition Department, Hospital Universitario de Castellón, Valencia, Spain; ^18^ Department of Medicine and Surgery, Universidad Cardenal Herrera-CEU, CEU Universities, Castellón, Spain; ^19^ Endocrinology & Nutrition Department, Hospital Universitario Virgen del Rocío, Sevilla, Spain; ^20^ Endocrinology & Nutrition Department, Hospital Regional Universitario de Málaga, Málaga, Spain; ^21^ Endocrinology & Nutrition Department, Hospital Universitario Puerta de Hierro, Madrid, Spain; ^22^ Endocrinology & Nutrition Department, Hospital Universitario Príncipe de Asturias, Madrid, Spain; ^23^ Endocrinology & Nutrition Department, Hospital Clínico San Carlos, Madrid, Spain; ^24^ Endocrinology & Nutrition Department, Hospital Universitario Clínico San Cecilio, Granada, Spain; ^25^ Endocrinology & Nutrition Department, Hospital Clinic de Barcelona, Barcelona, Spain; ^26^ Endocrinology & Nutrition Department, Hospital Universitario De Albacete, Albacete, Spain; ^27^ Endocrinology & Nutrition Department, Hospital de la Santa Creu i Sant Pau, IR-SANT PAU, CIBERER U747 (ISCIII), Barcelona, Spain; ^28^ Endocrinology & Nutrition Department, Hospital Universitario de Salamanca, Salamanca, Spain; ^29^ Endocrinology & Nutrition Department, Hospital Universitario La Princesa Madrid, Madrid, Spain; ^30^ Endocrinology & Nutrition Department, Hospital La Fe, Valencia, Spain; ^31^ Endocrinology & Nutrition Department, OSI Bilbao-Basurto, Hospital Universitario de Basurto, Bilbao, Spain; ^32^ University of the Basque Country UPV/EHU, Leioa, Spain; ^33^ Endocrinology & Nutrition Department, Hospital Universitario La Ribera, Valencia, Spain; ^34^ Department of Endocrinology, Diabetes and Metabolism, Coimbra Local Health Unity, Coimbra, Portugal; ^35^ Institute for Research and Innovation in Health of the University of Porto, Porto, Portugal; ^36^ Institute of Molecular Pathology and Immunology of the University of Porto, Porto, Portugal; ^37^ Coimbra Institute for Biomedical Imaging and Translational Research, Coimbra, Portugal; ^38^ Pituitary Tumor Unit, Endocrinology Department, Hospital CUF Descobertas, Lisbon, Portugal; ^39^ Faculdade de Medicina, Universidade Católica Portuguesa, Lisbon, Portugal; ^40^ Pathology Department, Hospital Universitario Vall de Hebrón, Barcelona, Spain; ^41^ Patholoy Department, Hospital Universitario Ramón y Cajal, Madrid, Spain; ^42^ Instituto de Investigación Biomédica Ramón y Cajal (IRYCIS), CIBERONC, Madrid, Spain; ^43^ Endocrinology & Nutrition Department, Hospital Universitario Germans Trias i Pujol, Cataluña, Spain; ^44^ CIBERER group 747, Cataluña, Spain

**Keywords:** acromegaly, somatostatin receptor ligands, surgical remission, growth hormone, prolactin co-secreting pituitary adenoma

## Abstract

**Aim:**

To evaluate which factors are associated with a higher probability of failure to surgical and first-generation somatostatin receptor ligands (fgSRLs) treatment in patients with growth hormone and prolactin co-secreting pituitary adenomas (GH&PRL-PAs).

**Methods:**

Acromegaly patients with GH&PRL-PAs included in the ACRO-SPAIN study were enrolled. GH&PRL-PAs were defined as tumors with serum PRL levels above the upper limit of normal and positive immunostaining for GH and PRL, or with PRL levels ≥100 ng/mL when immunostaining data were not available.

**Results:**

A total of 126 acromegaly patients with GH&PRL-PAs who underwent transsphenoidal pituitary surgery were included, and 42.1% (*n* = 53) were biochemically cured at the immediate postoperative evaluation. Knosp grade >2 (odds ratio (OR) 3.48, 95% CI 1.28–9.38), higher serum GH (OR 1.01, 95% CI 1.01–1.08) and IGF-1 (OR 1.60, 95% CI 1.05–2.45) levels were associated with a lower probability of surgical cure. Sixty-eight patients received first-line medical therapy as follows: fgSRLs in monotherapy (*n* = 22), fgSRL plus cabergoline (*n* = 37), cabergoline in monotherapy (*n* = 7) and pegvisomant in monotherapy (*n* = 2). Among the cases treated with fgSRL in monotherapy, 18.2% (*n* = 4/22) were resistant. We identified as predictors of fgSRL resistance (in monotherapy and combined with cabergoline) a Knosp grade >2 (OR 8.75, *P* = 0.003), high GH levels at acromegaly diagnosis (OR 1.02, *P* = 0.031) and higher postoperative GH levels (OR 1.05, *P* = 0.006), but no predictors of response to fgSRL in monotherapy were identified.

**Conclusion:**

The clinical predictors of surgical failure and of fgSRL resistance in patients with GH&PRL-PAs are similar to those described in acromegaly without PRL, co-secretion.

**Significance statement:**

In this article focused on GH&PRL pituitary adenomas, we found that a Knosp grade >2, and higher serum GH and IGF-1 levels were associated with a lower probability of surgical cure in these tumors. Regarding the response to fgSRL in monotherapy, 18% of the patients with GH&PRL pituitary adenomas were classified as resistant. Knosp grade >2 (OR 8.75, *P* = 0.003), high GH levels at acromegaly diagnosis (OR 1.02, *P* = 0.031), and higher postoperative GH levels (OR 1.05, *P* = 0.006) were predictors of non-response to fgSRL (monotherapy or combined with cabergoline), while no predictors of response to fgSRL in monotherapy were identified. Thus, we concluded the clinical predictors of surgical failure and of fgSRL resistance in patients with GH&PRL-PAs are similar to those described in acromegaly without PRL co-secretion.

## Introduction

Growth hormone (GH) and prolactin (PRL) co-secretion may be present in approximately 15–40% of all acromegaly cases, depending on the employed definition for co-secretion (1, 2, 3). The clinical presentation, tumor growth behavior and response to treatment of GH and PRL co-secreting pituitary adenomas (GH&PRL-PAs) can be heterogeneous. We recently reported that GH&PRL-PAs present at a younger age, are more often symptomatic, larger and more invasive in comparison to pure GH-secreting pituitary adenomas (GH-PAs) (3). Despite their higher invasiveness, we did not find worse surgical remission outcomes (3). However, we observed that the biochemical control achieved with preoperative first-generation somatostatin receptor ligands (fgSRLs) in monotherapy was significantly worse in the GH&PRL-PA group compared to patients with GH-PAs, supporting the inclusion of cabergoline in combination with fgSRLs as first-line medical treatment in this subset of patients, at least in the preoperative period (4). To the best of our knowledge, our series from the ACRO-SPAIN registry is the largest focused on studying the clinical, hormonal and radiological profile, and surgical and medical outcomes of these tumors. However, there are previous studies with fewer patients with GH&PRL-PA reporting a more aggressive behavior, a need for higher doses of medical therapy, and an overall worse prognosis for GH&PRL-PAs in comparison to GH-PAs (1, 2, 5). No previous study has specifically evaluated the factors associated with a lower rate of surgical remission and a higher rate of resistance to fgSRLs rate in patients with GH&PRL-PAs.

In this study, we aimed to provide a better understanding of the group of GH&PRL-PAs, and specifically evaluate whether there are any clinical, hormonal, radiological or pathological features that may be associated with a worse surgical outcome and a higher rate of resistance to fgSRLs.

## Methods

### Study population

ACRO-SPAIN is a multicenter retrospective registry of patients with acromegaly treated between 2003 and 2023 in 33 tertiary Spanish hospitals. At the time of the data analysis (20 December 2024), there were 685 patients included in the database. As we have previously described, the inclusion criteria of this study were the following: i) biochemical diagnosis of acromegaly established by clinical practice guidelines criteria; ii) available data of clinical, hormonal and radiological tumor characteristics preoperatively and postoperatively, and/or premedical and post-medical treatment and iii) follow-up data for longer than 3 months after surgery. Those patients who did not undergo pituitary surgery were excluded (3). In addition, for the current study, we included only acromegaly patients with GH&PRL-PAs. We defined GH&PRL-PAs as tumors with serum PRL levels above the upper limit of normal (ULN) and positive immunostaining for GH and PRL (*n* = 118), or when serum PRL levels were >100 ng/mL regardless of PRL immunostaining (*n* = 17), in patients with biochemically proven acromegaly. We excluded nine patients with acromegaly who did not undergo pituitary surgery. Other causes of secondary hyperprolactinemia were excluded (including hyperprolactinemia-inducing medications, renal insufficiency, uncompensated primary hypothyroidism and macroprolactinemia). Blood samples for PRL were extracted after at least 15–30 min of rest, avoiding the stress of venipuncture.

ACRO-SPAIN is an open registry maintained in RedCap® that collects comprehensive patient information including demographics, clinical characteristics, hormonal, radiological and pathological data, as well as treatment-related outcomes. Detailed information on ACRO-SPAIN is available in our previous studies (3, 4, 6, 7). Invasion was assessed by magnetic resonance imaging (MRI) using the Knosp classification. A pituitary adenoma was considered invasive if it had a Knosp grade of 3 or 4 (8).

The local ethical committee approved the study. The study was conducted according to the mandates of the Declaration of Helsinki and good clinical practice. Patient consent was waived due to the retrospective nature of the study. Informed consent was obtained only for patients who were followed up or prospectively enrolled.

### Measured variables and definitions

The diagnosis of acromegaly was made according to the recommendation of the acromegaly guidelines at the time of diagnosis (9, 10). Serum insulin-like growth factor-1 (IGF-1) and GH levels were measured locally in each center by immunochemiluminescence, immunoradiometric assay (IRMA) or electrochemiluminescence ‘ECLIA’ using different assays such as IMMULITE 2000, Liaison XL (Diasorin, Italy) or ImmuliteXP. Due to the variability in the IGF-1 assays, and to allow comparisons, the percentage above the ULN was used. GH and IGF-1 were measured in each patient at baseline, after surgery (immediate postoperative evaluation, 3–6 months after surgery), after at least 6 months of medical treatment, and at the last follow-up visit (long-term postoperative evaluation more than 12 months after surgery).

We defined surgical remission considering the biochemical status at least 3 months after surgery, using the Cortina definition (2000 criteria) (11) and the 2010 criteria of the AACE and Endocrine Society guidelines (10, 11). We classified patients as in biochemical remission when both criteria were met, i.e., normalization of IGF-I and random GH < 1 ng/mL.

### Surgical treatment outcomes and pathological information

A transsphenoidal approach was used in all surgeries. More details about surgical procedures and definitions of major and minor complications are available in our previous study (3). Standard H&E-stained sections and relevant immunochemistry staining sections were used for the histopathological diagnosis and classification of GH pituitary tumors. Information about cellular atypia, immunostaining pattern (for human GH, PRL, TSH, ACTH, FSH and LH) and Ki-67 were registered. In addition, the cytokeratin immunohistochemical pattern was reported in 47 cases, allowing their separation into densely granulated and sparsely granulated PAs (12). Furthermore, information about the subtype of pituitary tumor based on transcription factors and according to the 2022 WHO classification was available in 67 patients, and all of them were positive for PIT1 (13).

### Medical therapy outcomes

We selected patients treated with fgSRLs for at least 3 months, and with IGF-1 levels measured 3–6 months after surgery. For the classification of IGF-1 control with medical therapies, we used the following definitions (14): i) complete response: achieving IGF-1 levels within the normal range for age and sex as determined by local laboratory reference values; ii) partial response: a reduction of IGF-1 levels ≥50% from baseline but without IGF-1 normalization; and iii) poor response: a reduction in IGF-1 levels <50% after starting medical treatment at maximum tolerated doses. Patients with partial or poor responses were categorized as non-controlled or resistant cases, respectively. Only those patients under maximum tolerated doses of fgSRL were categorized as partial or poor responders.

We collected information about the duration of treatment, maximal doses and side effects. We evaluated the response to fgSRL in the postoperative period (treatment was started at least 3–6 months after surgery) and at the last follow-up visit, aiming to identify cases of resistance at the start of medical therapy and at the last follow-up visit.

### Statistical analysis

Statistical analysis was performed using STATA 15. Categorical variables were expressed as percentages and absolute values of the variable, and quantitative variables were expressed as mean ± standard deviation (SD) or median ± ranges, depending on whether the normality assumption was met. For the comparison of differences in continuous parameters between two subgroups, we used the Student’s *t*-tests and linear regression tests. The Chi-squared (Chi2) test was used to compare categorical data. Univariate logistic regression analysis was used to estimate odds ratio (OR), and a multivariate logistic regression model was used for the adjustment of the OR by potential confounding factors. The selection of potential predictors of non-cure and non-response to fgSRL was based on the data published in the previous literature in GH-PA. In addition, other potential variables such as PRL levels and mammosomatotroph histology were evaluated. In all cases, a two-tailed *P* value < 0.05 was considered statistically significant.

## Results

### Baseline characteristics

A total of 126 patients with GH&PRL-PAs were included. One hundred and eighteen had available and positive immunostaining for PRL, and the other eight cases with no available immunostaining had serum PRL levels above 100 ng/mL (median 160.8 ng/mL, range 144–3,100). The baseline characteristics of the whole cohort are described in Table 1. Two patients had hereditary acromegaly (familial isolated pituitary adenoma syndrome and multiple endocrine neoplasia 1 (MEN1) syndrome), and there were six patients with pituitary apoplexy as the presentation of acromegaly.

**Table 1:** Baseline characteristics of patients with GH&PRL pituitary adenomas and differences between pretreated and non-pretreated patients.

	Global cohort (*n* = 126)	Presurgical medical treatment (*n* = 58)	Direct pituitary surgery (*n* = 68)	*P* value
Age at diagnosis (years)	44.2 (range 11.3–73.8)	41.6 ± 13.9	44.9 ± 15.6	0.220
Female sex	60.3% (*n* = 76)	58.6% (*n* = 34)	61.7% (*n* = 42)	0.719
Type 2 diabetes	14.3% (*n* = 18)	22.4% (*n* = 13)	7.4% (*n* = 5)	0.016
Hypertension	25.4% (*n* = 32)	17.2% (*n* = 10)	32.4% (*n* = 22)	0.052
Headache	51.6% (*n* = 65)	48.3% (*n* = 28)	54.4% (*n* = 37)	0.492
Sleep apnea syndrome	22.2% (*n* = 28)	15.5% (*n* = 9)	27.9% (*n* = 19)	0.095
Visual impairment	27.4% (*n* = 34)	24.1% (*n* = 14)	32.4% (*n* = 22)	0.309
Macroadenomas	91.1% (*n* = 113)	91.4% (*n* = 53)	90.9% (*n* = 60)	0.927
Tumor size (mm)	19.0 (range 2.6–59.0)	20.2 ± 10.6	22.2 ± 13.0	0.360
Knosp grade >2	33.9% (*n* = 40/118)	35.7% (*n* = 20/56)	32.3% (*n* = 20/62)	0.692
T2-MRI hypointensity	34.9% (*n* = 30/86)	22.5% (*n* = 9/40)	45.7% (*n* = 21/46)	0.025
IGF-1 at diagnosis (SD ULN)	2.5 (range 1.2–6.4)	3.7 ± 7.6	2.6 ± 1.1	0.236
Random GH at diagnosis (ng/mL)	9.4 (range 1.5–149.0)	26.6 ± 49.7	12.8 ± 13.6	0.035
PRL at diagnosis (ng/mL)	99.6 (range 24–4,700)	310.0 ± 873.3	280.5 ± 1,035.9	0.867
Hypopituitarism at diagnosis	21.4% (*n* = 27)	24.1% (*n* = 14)	19.1% (*n* = 13)	0.494
ACTH deficit	8.7% (*n* = 11)	6.9% (*n* = 4)	10.3% (*n* = 7)	0.501
Secondary hypothyroidism	13.5% (*n* = 17)	10.3% (*n* = 6)	16.2% (*n* = 11)	0.340
Hypogonadotropic hypogonadism	31.8% (*n* = 40)	39.7% (*n* = 23)	25% (*n* = 17)	0.078
Densely granulated tumors (*n* = 47)	42.6% (*n* = 20/47)	36.7% (*n* = 8/22)	48% (*n* = 12/25)	0.421
Ki-67 > 3%	14.6% (*n* = 15/103)	12.2% (*n* = 6/49)	16.7% (*n* = 9/54)	0.525

GH, growth hormone; IGF-1, insulin-like growth hormone-1; MRI, magnetic resonance imaging; PRL, prolactin; SD, standard deviation; ULN, upper limit of normal.

All patients underwent transsphenoidal pituitary surgery. There were 58 out of the 126 patients (46.0%) treated preoperatively (i.e., received medical therapy before surgery): fgSRL in monotherapy (*n* = 30; 23.8%), fgSRL and cabergoline (*n* = 19; 5.1%), or cabergoline in monotherapy (*n* = 9; 7.1%). Patients pretreated with medical treatment had a higher prevalence of type 2 diabetes and lower prevalence of hypertension, higher GH levels at diagnosis, and more frequently harbored hypointense tumors than patients who did not receive medical therapy preoperatively. No other significant differences were found between the pretreated and non-pretreated groups (Table 1), either in the rates of surgical remission in the short-term follow-up (3–6 months after surgery) (36.2 vs 47.1%, *P* = 0.219) or in the long-term evaluation (50 vs 48.5%, *P* = 0.869).

In relation to the pathological subtypes of GH&PRL-PAs according to the WHO 2022 classification (*n* = 67), most of them were classified as mammosomatotroph (*n* = 50), followed by somatotroph (*n* = 8), lactotroph (*n* = 2) and mature or immature plurihormonal PIT1-lineage tumor (*n* = 7). The two cases with lactotroph tumors had IGF-1 levels 1.9 and 2.6 times above the ULN.

### Predictive factors of surgical failure

Of the 126 patients who underwent pituitary surgery, in the immediate postsurgical evaluation (3–6 months after surgery), 42.1% (*n* = 53) were biochemically cured. In six of these 53 patients with IGF-1 normalization (11.3%), hyperprolactinemia persisted in the postoperative period, with median levels of 38.4 (range 24–68) ng/dL. Three of these six cases normalized PRL levels spontaneously at the last follow-up visit, while in the other three patients, a tumoral rest was identified and hyperprolactinemia persisted in the following visits and were medically treated (cabergoline in monotherapy in one case with isolated hyperprolactinemia, and fgSRL in monotherapy in two patients with high IGF-1 and PRL levels).

**Figure 1:**
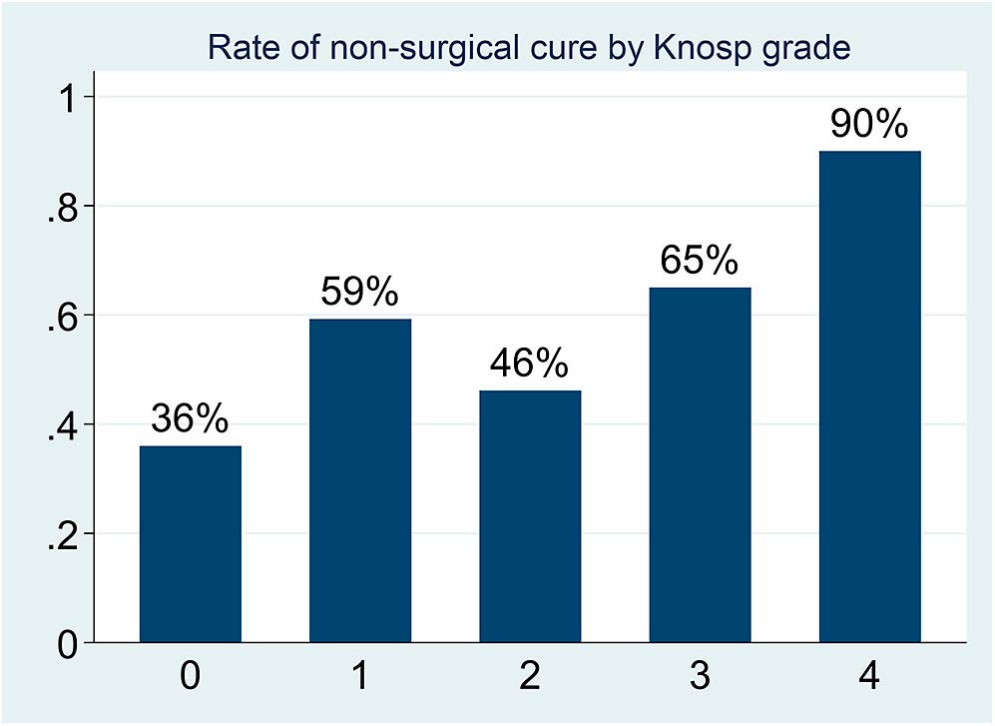
Probability of non-surgical cure depending on the Knosp grade.

There were 28 cases (22.2%) that experienced postsurgical vasopressin deficiency (nine cases with permanent deficiency), nine (7.1%) cerebrospinal leaks and three (2.4%) meningitis (two of them also had a cerebrospinal leak). Patients with a Knosp grade >2 and higher baseline serum GH and IGF-1 levels had a lower probability of achieving remission after surgery (Table 2). In the multivariate analysis, we tested whether these three variables were independently associated with surgical failure (Table 2), and the analysis confirmed that they were independent predictive factors. In addition, when we included the time of follow-up after surgery as a covariate, these three variables maintained their significance as predictors of surgical failure (Knosp ≥2 (OR 3.23, 95% CI 1.19–8.83), IGF1 (OR 1.58, 95% CI 1.03–2.41) and GH (OR 1.04, 95% CI 1.01–1.08)). As expected, the rate of surgical failure increased with an increasing Knosp grade (Fig. 1).

**Table 2:** Factors associated with a lower rate of surgical cure (immediate surgical cure) in GH&PRL co-secreting pituitary adenomas.

	Odds ratio (95%CI) univariate	*P* value	Odds ratio (95%CI) multivariate
Age at diagnosis (years)	0.98 (0.96–1.00)	0.105	
Female sex	1.14 (0.55–2.35)	0.721	
Type 2 diabetes	1.54 (0.54–4.41)	0.413	
Hypertension	0.65 (0.29–1.45)	0.294	
Headache	1.36 (0.67–2.76)	0.398	
Visual impairment	1.68 (0.75–3.75)	0.206	
Tumor size (mm)	1.02 (0.99–1.05)	0.264	
Knosp grade >2 (*n* = 118)	3.82 (1.61–9.07)	0.001	3.48 (1.28–9.38)
T2-MRI hypointensity (*n* = 86)	0.61 (0.25–1.49)	0.277	
Presurgical medical therapy	1.56 (0.76–3.21)	0.218	
IGF-1 at diagnosis (SD ULN)	1.75 (1.21–2.54)	<0.001	1.60 (1.05–2.45)
GH at diagnosis (μg/mL)	1.06 (1.02–1.10)	<0.001	1.04 (1.01–1.08)
PRL at diagnosis (ng/mL)	1.00 (1.00–1.00)	0.418	
Hypopituitarism	1.07 (0.45–2.55)	0.875	
Mammosomatotroph	1.50 (0.51–4.43)	0.463	

GH, growth hormone; IGF-1, insulin-like growth hormone-1; MRI, magnetic resonance imaging; PRL, prolactin; SD, standard deviation; ULN, upper limit of normal.

### Predictive factors of resistance to medical therapy

**Figure 2:**
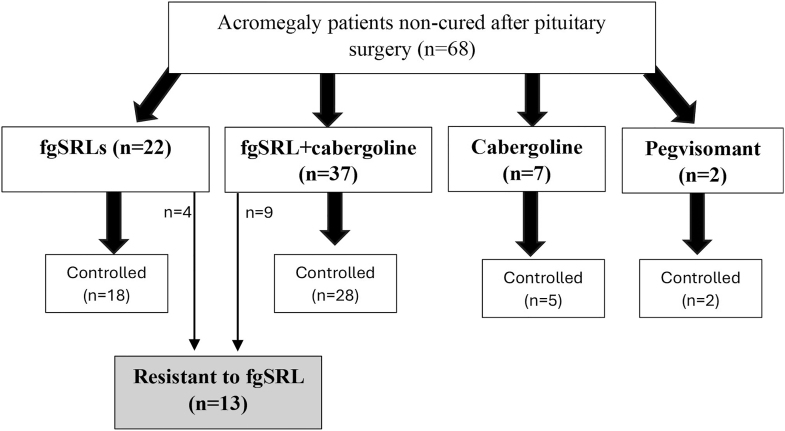
Postoperative medical treatments employed in non-cured acromegaly patients. fgSRL = first generation somatostatin receptor ligands.

Medical therapy for patients with active disease or those who relapsed during follow-up included fgSRLs as monotherapy in 22 patients (13 lanreotide and nine octreotide), fgSRLs in combination with cabergoline in 37 patients, cabergoline as monotherapy in seven patients and pegvisomant in monotherapy in two cases (Fig. 2). The median doses employed were 120 mg/month (range 60–120) for lanreotide, 30 mg/month (range 20–60) for octreotide and 1.5 mg/week (range 0.5–8) for cabergoline. There was one patient classified as non-cured treated with radiotherapy, and the other six cases were followed without active treatment. Biochemical control was achieved in 81.8% (*n* = 18) of patients treated with fgSRL alone, 72.7% (*n* = 28) of those treated with fgSRL and cabergoline, and 71.4% (*n* = 5) of those treated with cabergoline in monotherapy. The median doses of lanreotide, octreotide and cabergoline were 120 mg/month (range 30–120), 30 mg/month (range 10–60) and 2 mg/week (range 0.5–8), respectively.

Among all operated patients treated with fgSRL at maximum therapeutic doses, either alone or in combination with cabergoline, 22% (*n* = 13/59) did not respond and were classified as resistant cases. When we analyzed the predictors of non-response to fgSRL in the group of patients treated with fgSRL in monotherapy, 18.2% (*n* = 4/22) did not respond. No predictors of response for fgSRL in monotherapy were identified (Table 3). However, when we analyzed the predictors of response for patients treated with fgSRL in monotherapy or in combination with cabergoline, we found that a Knosp grade >2 (OR 8.75 (1.70–45.01), *P* = 0.003), high GH levels at diagnosis (OR 1.02 (1.00–1.03), *P* = 0.031), and higher postoperative GH levels (OR 1.00 (1.00–1.10), *P* = 0.006) were predictors of non-response. In relation to predictors of non-response only in the group treated with fgSRL in combination with cabergoline, similar predictors to those analyzed for both fgSRL in monotherapy and in combination with cabergoline were identified (Knosp grade >2 (OR 12.6 (1.35–117.57), *P* = 0.007), and higher postoperative GH levels (OR 1.05 (1.00–1.12), *P* = 0.032).

**Table 3:** Predictive factors of resistance to fgSRL (used in monotherapy) in GH&PRL co-secreting pituitary adenomas.

	Odds ratio (95%CI) univariate	*P* value
Age at diagnosis (years)	0.93 (0.85–1.03)	0.138
Male sex	3.75 (0.32–43.31)	0.260
Hypertension	0.67 (0.06–7.85)	0.742
Sleep apnea syndrome	0.87 (0.07–10.42)	0.910
Tumor size (mm)	1.01 (0.89–1.15)	0.880
Knosp grade >2 (*n* = 51)	5.0 (0.42–59.66)	0.173
T2-MRI hypointensity (*n* = 37)	2.67 (0.12–57.62)	0.535
Presurgical medical therapy	1.57 (0.18–13.86)	0.685
IGF-1 at diagnosis (SD ULN)	2.52 (0.75–8.42)	0.096
Postsurgical IGF-1 (SD ULN)	0.98 (0.86–1.13)	0.637
GH at diagnosis (ng/mL)	1.03 (1.00–1.08)	0.049
Postsurgical GH (ng/mL)	1.05 (0.98–1.12)	0.083
PRL at diagnosis (ng/mL)	0.99 (0.97–1.01)	0.276
Densely granulated tumors (*n* = 7)	4.00 (0.12–136.96)	0.439

GH, growth hormone; IGF-1, insulin-like growth hormone-1; MRI, magnetic resonance imaging; PRL, prolactin; SD, standard deviation; ULN, upper limit of normal.

When we evaluated the response to fgSRL at the last follow-up visit in those patients treated with fgSRL in monotherapy or in combination with cabergoline in the immediate postoperative period, after a median follow-up of 6 years (1–18), 20.4% (*n* = 12) had active disease (elevated IGF-1). Of the other 47 (37.3%) patients with controlled disease, six (4.8%) patients who had received radiotherapy were cured with no need of medical treatment, 23 (18.3%) continued under treatment with fgSRL in monotherapy or in combination with cabergoline (one of them was reoperated), five (4.0%) with fgSRL and radiotherapy, five (4.0%) were treated with pegvisomant in monotherapy (four had received radiotherapy and one was reoperated), five (4.0%) with pegvisomant and fgSRL, one (0.8%) with pegvisomant and pasireotide and two (1.6%) with pasireotide in monotherapy. Thus, overall, only 23 out of the initial 59 cases (39%) treated with fgSRL alone or in combination with cabergoline maintained controlled acromegaly, while the other cases required additional treatments.

At the last follow-up visit, a total of 12 patients (9.5%) experienced recurrence of acromegaly. The median time from the first surgery to recurrence was 3.5 years (range 1–16).

## Discussion

Our study is the first series focusing on the identification of predictive factors for surgical failure and fgSRL resistance in this specific group of GH&PRL-PAs. The main findings of our study were that immediate postsurgical remission was achieved in 42% of patients and the probability of cure was lower in patients with a Knosp grade >2 with higher GH and IGF-1 levels at diagnosis. Regarding fgSRLs, up to 18% of patients treated with fgSRL in monotherapy were resistant to fgSRL when the evaluation was performed in the immediate postoperative period. It should be highlighted that when we evaluated the proportion of patients with the rate of biochemical control with fgSRL alone or in combination with cabergoline in the last follow-up visit, the rate decreased to 39%.

In our series we observed that presurgical medical therapy was more commonly used in patients with type 2 diabetes and hypertension and with higher GH levels at diagnosis. This observation is probably explained by the fact that presurgical medical treatment is frequently employed for the most symptomatic acromegaly cases. Although preoperative medical treatment is not currently a general recommendation (15, 16), this practice might be worthy for those patients with a higher surgical risk, especially if they have severe pharyngeal thickness, sleep apnea, high-output heart failure or when the delay from diagnosis to surgery is more than 3–6 months (10). However, in our cohort there were no differences in the rate of surgical cure between pretreated and non-pretreated patients either in the short- or long-term follow-up. Although in most patients preoperative SRLs reduce GH and IGF-1 levels, promote tumor shrinkage and soften tumor consistency (17), our results, in accordance with previous studies (18, 19), also described non-significant differences between patients pretreated and non-pretreated with fgSRL in terms of surgical remission. Pita-Gutierrez *et al.* in a meta-analysis (18) found no significant difference in the cure rate between pretreatment and direct surgery groups (OR 1.62, 95% CI 0.93–2.82) supporting that surgical cure or non-remission is influenced by other factors regardless of the clinical benefits or symptom amelioration of medical treatment. In Zhang *et al.* meta-analysis (19) there were also no significant differences in long-term surgical remission between medically pretreated and non-pretreated patients (RR 1.03, 95% CI 0.86–1.24). Nevertheless, another recent meta-analysis encompassing 688 patients found that the rate of short-term biochemical remission was higher in the group pretreated with fgSRL compared to non-pretreated patients (50.5 vs 35.1%; OR 2.07, 95% CI 1.50–2.87) (19). However, no previous study has evaluated the impact of fgSRL on surgical remission rate in the subgroup of GH&PRL-PAs. Our data regarding preoperative medical therapy supports the adoption of a similar approach as recommended for pure GH-secreting PAs. The use of preoperative medical therapy for GH&PRL-PAs should not be recommended with the aim of improving biochemical control after surgery but it may be considered to ameliorate clinical symptoms before surgery. The only previous study evaluating response to fgSRL in GH&PRL-PAs in non-operated patients is from the ACRO-SPAIN register. In this study we observed that after 6 months of treatment, in the group of patients under fgSRL as monotherapy, those patients with GH&PRL-PAs had worse control compared to GH-PAs (29.4 vs 55.1%, *P* = 0.04) when receiving fgSRL in monotherapy, but these differences disappeared when both received combination treatment with fgSRL and cabergoline (4). For this reason, we propose that in GH&PRL-PAs cosecreting tumors, the combination of fgSRL and cabergoline for the preoperative treatment should be considered instead of fgSRL in monotherapy. This practice is in line with our philosophy in the treatment of acromegaly: emphasizing personalized therapy as this approach allows a greater number of patients to achieve control within a shorter period of time (20). In this regard, we also found that presurgical fgSRL was prescribed more frequently in patients harboring T2-hypointense tumors on MRI, which is a feature associated with a higher likelihood of response as hypointense tumors are more often densely granulated tumors and have a higher expression of type 2 somatostatin receptors (21, 22). Nonetheless, we must emphasize that in our study, in the postoperative period only 37 out of the 68 cases were treated with the combination of cabergoline and fgSRLs. This may be related to the fact that we did not find significant differences between fgSRL in monotherapy and cabergoline plus fgSRL in relation to the rate of biochemical control in the postoperative period.

In relation to acromegaly recurrence, we reported a rate of 9.5% with a median time since the first surgery and recurrence of 3.5 years. This rate is a little higher than that reported by other authors such as Losa M who found recurrence only in 3.4% of the patients (23). The mean time of recurrence was in line with that reported in the Maroufi SF meta-analysis (4.16 years after the initial treatment) (24). Nonetheless, they described recurrence of acromegaly only in 2–3% of the patients. Thus, it is possible that GH&PRL-PAs have a more aggressive behavior than pure GH-PAs.

In our study, surgical cure was achieved in 42.1% of the patients. The surgical cure rate reported in other series of GH&PRL-PAs ranges from 20 to 70% (5, 24). In a study encompassing 182 GH-PAs and 97 GH&PRL-PAs, the surgical cure rate was similar in GH-PAs and GH&PRL-PAs (68.4 vs 59.7%; *P* = 0.187) (25). In contrast, a smaller series including (5) 69 GH-PAs and 22 GH&PRL-PAs reported a lower surgical cure in the GH&PRL co-secreting group (20 vs 68%; *P* = 0.01). In addition, it is interesting to point out that the rate of postsurgical hyperprolactinemia was five times greater in the group of GH&PRL-PAs than in GH-PAs and this may be an indicator of tumoral persistence in cosecreting tumors (3). In our study, there were 10% of the cases classified as cured based on serum IGF-1 levels that had persistent hyperprolactinemia. Thus, if we consider the combined criteria of normal IGF-1 and PRL for defining surgical remission in GH&PRL-PAs, the proportion of remission will be lower than that commonly described in GH-PAs. Nonetheless, postsurgical hyperprolactinemia may also be related to compression or displacement of the pituitary stalk, limiting the applicability of this criterion especially in the immediate postoperative period. These studies are probably not comparable since the neurosurgeon team, tumor invasiveness characteristics and the biochemical remission criteria are different across the series.

Consistent with findings from previous series of acromegaly with pure GH-PAs, our study revealed that patients with GH&PRL-PAs who had a Knosp grade >2 along with higher serum GH and IGF-1 levels had an increased probability of surgical failure. Among these, Knosp grade is one of the most influential risk factors of surgical failure in acromegaly (26, 27, 28, 29, 30, 31, 32, 33, 34), as well as for other pituitary tumors (35).

Presurgical GH and IGF-1 levels have been described as important predictive factors of surgical cure/non-cure in GH-PAs in most series (27, 29, 31, 33, 34, 36). Several other risk factors have been identified including the dual staining for GH&PRL compared to single GH staining (37). Dehghan *et al.* described a 1.5-fold higher postoperative remission rate in the single staining adenomas than in dual staining counterparts, but the differences were not significant likely due to the small size of the cohort (37).

We reported that 18% of the GH&PRL-PA cases treated with fgSRL in monotherapy did not achieve complete IGF-1 normalization and were classified as resistant to fgSRLs. Nevertheless, the rate of resistance increased up to 60% when we reassessed the acromegaly situation in the last follow-up visit since only 39% of the patients who were initially treated with fgSRL in monotherapy or combination with cabergoline maintained an adequate IGF-1 control with no need for additional therapies. Resistance to fgSRL is common in clinical practice, occurring in 30% up to 60% of patients according to some studies (38, 39). These proportions are variable depending on the definition used for biochemical control. For example, the classic definition proposed by Colao (14) indicated that both GH and IGF-1 levels should be normal for considering complete response to fgSRL, while others propose that IGF-1 is the standard of care in clinical practice and a normal value independently of GH levels should be classified as controlled (40). In addition, recent guidelines suggest as acceptable target IGF-1 levels up to 1.3 × ULN (16, 41). It is important to emphasize that in our previous study evaluating the response to fgSRL in the preoperative period, the rate of resistance to fgSRL was higher in GH&PRL-PAs than in pure GH-PAs (55.1 vs 29.4%, *P* = 0.04), but no differences were detected between both groups in the postoperative period in our current study. In this regard, it is very likely that the debulking surgery effect mitigates these differences (4). Thus, it is important to highlight that in our series GH&PRL-PAs were larger and tended to be more invasive at diagnosis and both factors tended to be associated with resistance to fgSRL (42, 43). Nonetheless, we did not confirm the role of the initial tumor size as a predictor of tumor resistance, probably due to the debulking effect of surgery. Thus, it is important to consider that the response to fgSRL is not totally comparable in the preoperative and postoperative periods, since some patients initially resistant to fgSRLs may become responsive if a significant tumor debulking is performed, especially in patients with large tumors.

In our study, 71% (five out of seven cases) of the patients with GH&PRL-PAs treated with cabergoline in monotherapy achieved normal IGF-1 levels. This good response rate, higher than previous studies (44, 45), could be explained because patients with GH&PRL-PAs commencing cabergoline monotherapy had milder IGF-1 elevation than patients with GH&PRL-PAs receiving fgSRLs or patients with pure GH-PAs (46). However, our data should be cautiously interpreted due to the lower number of patients. Abs *et al.* evaluated the effect of long-term cabergoline monotherapy in 64 acromegaly patients, including 16 GH&PRL-PAs. IGF-1 normalization was achieved in 35% of pure GH-PAs cases and in 50% of GH&PRL-PA patients (cabergoline dose ranging between 1.0 and 1.75 mg/week), where responding patients tended to have lower baseline IGF-1 (44). Later, a meta-analysis including 160 patients with acromegaly treated with cabergoline as monotherapy reported that 34% of them achieved normal IGF-1 levels (mean cabergoline dose was 2.6 mg/week), and hormonal remission was predicted by lower IGF-1 levels and high PRL levels at baseline (45). The best predictor for achieving hormonal remission with cabergoline monotherapy is a low baseline IGF-1, with an IGF-1 baseline concentration below 1.5 × ULN improving the likelihood for IGF-1 normalization. Importantly, cabergoline may also reduce tumor size in up to a third of patients (45), and complete shrinkage of mixed GH&PRL-PAs has also been reported for bromocriptine in monotherapy (47). Thus, cabergoline monotherapy can be considered as first-line medical therapy mainly for acromegaly patients with pure or cosecreting PAs with mild disease activity following surgery (48).

Like surgery, the main predictor of resistance to medical therapy (fgSRL in monotherapy or in combination with cabergoline) was having a pituitary tumor with Knosp grade >2. Several predictors of fgSRL resistance have been described previously and include male sex, younger age, initial GH and IGF-1 levels, high tumor volume, tumor hyperintensity on T2-weighted MRI and the expression of SSTR subtypes, among others (43). Although Knosp grade is not a widely accepted predictor of fgSRL response, the biological implications of invasion and their association with treatment outcomes have also been described (49). In relation to GH and IGF-1 levels as variables associated with the probability of response to fgSRLs, other studies have described similar results (50, 51). Our study supports that the variables linked to resistance to fgSRL in GH&PRL-PAs are similar to those described for pure GH-PAs; thus, the algorithm of personalized medicine is also applicable to these pituitary tumors (20), including the association of fgSRL with cabergoline rather than fgSRL in monotherapy as first-line therapy, especially in the preoperative period (4). Nevertheless, future studies focused on the treatment response and molecular profiling of GH&PRL-PAs are needed to confirm whether the molecular/biological background of these tumors is similar to the GH-PAs group. The prognostic value of immunohistochemistry biomarkers such as estrogen receptors (52) in GH&PRL-PAs, also requires clarification and the special case of multilineage tumors may provide relevant pathophysiological clues to the understanding of mixed tumors (53). Taken together, these data indicate that diagnosing and individualizing the treatment of acromegaly require integrating molecular, biochemical, clinical, radiological and histopathological information.

Our study has some limitations. Due to its retrospective design, potential bias during data collection might exist. However, patients with missing values in the main variables of interest were not included in the study and a strict protocol was followed for data inclusion and patient selection. Another limitation is not knowing the genetic background of the whole cohort, which may determine resistance, and not having all cases studied for cytokeratin pattern. In addition, the study was conducted in several centers using different IGF-1 and GH assays, which can differ significantly from each other. However, we mitigated this limitation by expressing biochemical data as for deviation above the ULN. In addition, there was missing information for histopathological data, as information on pituitary tumor subtype according to the WHO 2022 classification was available in only 50% of the included cases, which reduced the power to detect differences according to this variable. Information on dense and sparsely granular pattern was also missing in 60% of the cohort. Strengths of our study included the large sample size, being the largest series including GH&PRL-PAs attempting to find predictive markers of therapeutic failure (both surgical and medical failure) and the use of a comprehensive multivariable-adjusted logistic regression model to assess the statistical significance of several therapeutic predictive factors.

## Conclusion

Our study shows that the predictors of surgical failure and fgSRLs resistance in patients with GH&PRL-PAs are similar to those observed in acromegaly with GH-PAs, including Knosp grade and serum GH and IGF-1 levels at diagnosis as key factors. This comprehensive study advances our understanding of GH&PRL-Pas and these results reinforce the importance of individualized treatment strategies, integrating tumor characteristics and patient-specific factors to optimize clinical outcomes. Future studies aimed at deepening into the molecular characteristics and the different responses to medical treatments are warranted.

## Declaration of interest

MAC received speakers’ honoraria and consulting fees from Pfizer and Recordati. The other authors declare no conflict of interest.

## Funding

This work was funded by Sociedad Española de Endocrinología y Nutrición (SEEN): ‘Impacto de la co-secreción de prolactina en la expresión de marcadores moleculares y en la respuesta al tratamiento con análogos de somatostatina y agonistas dopaminérgicos en pacientes con acromegalia’.

## Institutional review board statement

The study was conducted according to the guidelines of the Declaration of Helsinki and approved by the Ethics Committee of the Hospital Universitario Ramón y Cajal, Madrid, Spain (approval date: 2nd June 2023, code: ACTA 454) and in each collaborating center.

## Informed consent statement

Patient consent was waived due to the retrospective nature of the study. Informed consent was requested only for patients who continued follow-up or who were prospectively included.
